# Does systemic lupus erythematosus increase the risk of complications from total hip arthroplasty?

**DOI:** 10.1186/s12891-021-04316-3

**Published:** 2021-05-19

**Authors:** Yongrui Cai, Zichuan Ding, Xiao Rong, Zong Ke Zhou

**Affiliations:** Department of Orthopedics, Research institute of Orthopedics, West China Hospital/West China School of Medicine, Sichuan University, 37# Wuhou Guoxue Road, Chengdu, PR China

## Abstract

**Background:**

Patients with systemic lupus erythematosus are more likely to receive THA than the general population. However, it is controversial whether SLE increases the risk of complications from THA. The purpose of this retrospective study was to reassess the risks from THA in patients with SLE under the management model of enhanced recovery after surgery.

**Methods:**

Patients with systemic lupus erythematosus diagnosed from December 2011 to December 2017 and treated with THA were compared with THA patients with osteoarthritis. The data were extracted from the medical record system of our department. The chi-square test and t-test were used for comparison.

**Results:**

The postoperative blood loss in patients with SLE was significantly higher than that in the control group, and the postoperative hemoglobin (Hb) and hematocrit (Hct) in the control group were lower than those in the control group (*P* < 0.05). There was no significant difference in the rate of blood transfusion (9.733 vs 8.133 *P* = 0.3148) or other complications between the two groups (*P* > 0.05).

**Conclusion:**

Well-controlled and well-managed SLE will not increase the risk of complications in THA, but can increase the amount of perioperative blood loss. Therefore, perioperative blood management is still essential in SLE patients.

## Introduction

Systemic lupus erythematosus (SLE) is a chronic autoimmune disease that can affect a variety of systems including bones and joints [1]. It is worth mentioning that due to the improvement of treatment, patients with SLE can have a longer survival time, so more patients will have musculoskeletal complications [2]. It has been reported that approximately 10% of SLE patients will develop osteonecrosis of the femoral head [3]. The specific mechanism of avascular necrosis of the femoral head in patients with SLE is not clear, and it is generally considered to be closely related to the long-term use of steroids, microvascular thrombosis and vasculitis [4, 5]. There are different treatments for osteonecrosis of the femoral head, but total hip arthroplasty (THA) is still considered to be the most effective method [6]. With the extension of the survival time of SLE patients, an increasing number of SLE patients will eventually undergo THA [7].

Although it is generally believed that patients with systemic lupus erythematosus could have good final function after THA, controversies remain with regard to whether patients with lupus have more complications after undergoing THA. Some case-control studies have shown that SLE patients have a higher incidence of perioperative complications and longer hospital stay during the perioperative period of total hip arthroplasty than patients with rheumatoid arthritis or osteoarthritis [8–14]. Some studies have reported that SLE is not an independent risk factor for perioperative adverse events in THA. The purpose of this study was to review the clinical outcomes of lupus patients who underwent THA at our institution.

## Method

### Patients selection

This study was conducted at West China Hospital, Sichuan University. Patients with a diagnosis of systemic lupus erythematosus who underwent total hip arthroplasty between December 2011 and December 2017 were included in our study and underwent THA (SLE group). As a control, we selected patients with osteoarthritis secondary to DDH who underwent THA in our hospital during the same period, matched 1:1 with the SLE group (Control group). All patients were informed of the specific treatment plan and the risks and signed an informed consent form. In addition, since our institution is a teaching hospital, all patients were informed and agreed to our access of their medical records and the use of related materials. Patients who met the following conditions would be excluded: (1) patients who did not undergo primary joint arthroplasty, (2) patients who underwent simultaneous bilateral surgery, and (3) patients with severe or uncontrolled comorbidities. Initially, our study included 54 SLE patients, but 2 patients refused to follow up, and 7 patients could not be followed up due to changes in contact information or contact address. Finally, we included a total of 45 SLE patients. In the matching process, the first factor we matched was the date of hospitalization (accurate to the month). Second, considering that systemic lupus erythematosus is more common in female patients, and that sex has a significant impact on indicators such as hemoglobin and platelets, we matched the sec of the two groups of patients. Finally, we narrowed the age gap between the two groups as much as possible within the range of choice. Finally, the 1:1 pairing of this study was completed. Accordingly, 45 patients who underwent THA in the same period were included in the control group.

### Perioperative management

All patients underwent the same preoperative examination after admission, and corresponding treatment measures were taken according to different preoperative symptoms. All patients were optimally prepared before surgery. All the patients in the SLE group were treated with low-dose steroids to control the disease (2.5–10 mg/qd) before operation, and continued to take the drug dose according to the physician’s original prescription.

All patients were treated with a posterolateral hip incision under general anesthesia. Tranexamic acid was given intravenously to 10 min before skin incision and 10 mg/kg was injected locally when the incision was closed. Tranexamic acid was administered at 10 mg/kg, 3 h and 6 h after the operation to reduce the amount of perioperative bleeding. All patients received noncement prostheses. The standard of blood transfusion was hemoglobin (Hb) < 70 or 70–100 g/L with symptomatic anemia (defined as severe mental status changes, palpitations and/or pallor).

### Clinical data

The indicators included the patient’s baseline demographic characteristics (age, sex, height, weight and body mass index), American Society of Anesthesiologists rating, coexisting diseases, preoperative HHS score, preoperative hematological indicators (hemoglobin(Hb), albumin(Ab), platelets(Plt), hematocrit(Hct), ESR, C-reactive protein(CRP)), intraoperative dominant bleeding, postoperative hematological indicators (Hb, Ab, Plt, Hct), perioperative adverse events, total blood loss, length of stay (LOS), HHS score and SF-12 score at the last follow-up.

Total blood loss was calculated by the Gross equation, and perioperative adverse events were recorded by hospital records and evaluated by two independent observers. Only postoperative complications, which that did not exist before the surgery, were included. The last telephone follow-up and imaging evaluation were conducted by two independent observers who did not participate in the operation or perioperative management.

### Statistical analysis

Quantitative data are given in the form of means. Qualitative data are presented as frequencies and percentages. The continuous variables were compared by Student’s t-test, and the qualitative comparison variables were compared by Pearson’s chi-square test or Fisher’s exact test. We use SPSS for Windows (version 21. 0 IBM Corp, Armonk, NY, USA) to perform all the statistical analyses.

## Result

The preoperative baseline characteristics of the two groups were similar except for age and BMI, and the results are shown in Table 1.

**Table 1:** Baseline characteristics

Characteristic	SLE	Control	*p*
Age (years)	40.78	46.91	0.0063
Female	88.67	88.67	> 0.9999
Height(m)	1.594	1.578	0.2491
Weight (kg)	53.42	57.71	0.0116
BMI	21	23.34	0.001
ASA	2.067	1.756	0.0437
HHS score	40.22	40.87	0.6453
Hemoglobin(g/L)	120.8	133.6	< 0.0001
Hematocrit(L/L)	0.3654	0.4067	< 0.0001
Platelet(10^9/L)	160.7	177	0.0977
Albumin(g/L)	40.06	42.25	0.0119
Follow-up time (years)	64.44	58.64	0.2362

The patients in the SLE group had more coexisting diseases than those in the control group, but there was no significant difference. The preoperative hemoglobin and hematocrit in the SLE group were significantly lower than those in the control group. There was no significant difference in other preoperative indexes (Table 2).

**Table 2:** Comorbidities Values are n (%) unless otherwise specified

	SLE	Control	*P* value
Hypertension	3 (6.67)	7 (15.56)	0.3148
Renal insufficiency	6 (13.33)	2 (4.44)	0.2663
Liver disease	3 (6.67)	1 (2.22)	0.6164
Pulmonary interstitial fibrosis	2 (4.44)	0 (0)	0.4944
Chronic bronchitis	1 (2.22)	0 (0)	> 0.9999
Asymptomatic bacteriuria	3 (6.67)	0 (0)	0.2416
Hypothyroidism	1 (2.22)	0 (0)	> 0.9999
Sjogren	1 (2.22)	0 (0)	> 0.9999
Atherosclerosis	0 (0)	3 (6.67)	0.2416
Total	20 (44.44)	13 (28.89)	0.189

The postoperative blood-related results and LOS of the two groups are shown in Table 3. The blood loss volume at POD1 and the TBL in the SLE group were significantly higher than those in the control group, but there was no significant difference in IBL. Similarly, the Hb and Hct at POD1 and POD3 in the SLE group were lower than those in the control group. Compared with the control group, there was no significant difference in the incidence of blood transfusion in the SLE group. In addition, the analysis based on steroid use showed that there was no significant difference in blood loss among patients with different doses (Table 4). The hospitalization time of the SLE group was longer than that of the control group, but there was no significant difference in other indexes.

**Table 3:** Hematological index after surgery

	SLE	Control	*P* value
Hb POD1(g/L)	104.4	116.9	<0.0001
Hb POD3(g/L)	91.71	106.5	<0.0001
HCT POD1(L/L)	0.3136	0.3538	<0.0001
HCT POD3(L/L)	0.2676	0.3171	<0.0001
Intraoperative blood loss (mL)	139.8	159.8	0.1647
Blood loss POD1(mL)	624.1	535.6	0.0471
Total blood loss (mL)	927	772.1	0.0054
Blood transfusion rate (mL)	15.56	6.67	0.3148
Albumin(g/L)	33.18	33.84	0.328
Platelet(10^9/L)	136.6	152.4	0.0756
Albumin transfusion rate(%)	17.78	6.67	0.1966
Day in hospital (days)	9.733	8.133	<0.0001

**Table 4:** different steroids use and total blood loss

Steroids use (mg/qd)	2.5	5	7.5	10	*P* value
Total blood loss (ml)	1044.455	931.905	789.9986	1244.566	0.1848

The comparison of perioperative adverse events is shown in Table 5. There was no significant difference in the overall incidence of adverse events between the two groups (*p* = 0.0743).

**Table 5:** Adverse event in hospital. Values are n (%) unless otherwise specified

	SLE	Control	*P* value
Ecchymosis	2 (4.44)	0 (0.00)	0.4944
Wound infection	2 (4.44)	0 (0.00)	0.4944
Wound fat liquefaction	2 (4.44)	0 (0.00)	0.4944
Wound swelling	0 (0)	6 (13.33)	0.0262
Hypocalcemia	1 (2.22)	0 (0.00)	> 0.9999
Hypoproteinemia	8 (17.78)	4 (8.89)	0.3529
Hypokalemia	6 (13.3)	1 (2.22)	0.1103
Anemia	7 (15.6)	2 (4.44)	0.1572
Vomiting	2 (4.44)	0 (0.00)	0.4944
Fever	2 (4.44)	0 (0.00)	0.4944
Pulmonary infection	1 (2.22)	1 (2.22)	> 0.9999
Deep vein thrombosis	0 (0)	0 (0)	> 0.9999
Pulmonary embolism	0 (0)	0 (0)	> 0.9999

From the preoperative evaluation to the last follow-up (Table 6), the HHS scores of the two groups were greatly improved, and there was no significant difference between the two groups. At the last follow-up, the SF-12 score of the SLE group was lower than that of the control group (t-test *p* = 0.006). There were no long-term prosthesis-related complications in either group.

**Table 6:** Mid-term outcomes. Values are n (%) unless otherwise specified

	SLE	Control	*P* value
HHS	89.56	90.16	0.6453
SF-12	48.91	52.38	0.006
DVT	0	0	> 0.9999
PE	0	0	> 0.9999
Dislocation	4 (8.89)	2 (4.44)	0.6766
Infection	0	0	> 0.9999
Periprosthetic fracture	0	0	> 0.9999
Revision	0	0	> 0.9999
Aseptic loosening	0	0	> 0.9999

## Discussion

Our study reveals that the risk of THA in SLE patients is not obviously increased under continuously optimized perioperative management and surgical techniques. Notably, in our cohort, SLE patients’ general preoperative condition was worse than that of patients with matched OA, including lower hemoglobin, hematocrit, and albumin levels and higher inflammatory markers, and ASA classification. The only significant differences between these two types of patients in perioperative and mid-term outcomes were in total blood loss and length of stay.

Our study shows that the perioperative risk of bleeding in SLE patients remains a concern. Earlier research on SLE has suggested that SLE itself may give rise to antibodies to anticoagulation factors and abnormal platelet activation and aggregation [15, 16]. These predispose patients with SLE to coagulation disorders and maybe also a higher risk of bleeding and thrombosis [17, 18]. In line with our findings, an earlier investigation discovered that hemoglobin was lower in SLE patients than in other patients, which also pointed to higher perioperative blood loss in SLE patients than in other patients [19]. However, unlike other studies, ours did not find a different blood transfusion rate between the groups. This may be associated with the more aggressive preoperative preparation and the application of tranexamic acid. Other studies have shown that not only patients with good basic conditions and fewer coexisting diseases but also patients with bleeding tendencies or blood coagulation disorders similar to SLE can also benefit from the use of TXA [20–23]. Therefore, we have reason to think that TXA can also reduce the perioperative blood loss of patients with SLE, thus reducing their blood transfusion rate. Steroids are a powerful set of drugs for controlling SLE. Whether the use of steroids increases the risk of perioperative adverse events remains controversial [24, 25]. Our study did not find that there were significant differences in perioperative adverse events among patients treated with different doses of steroids. These results suggest that it is safe for patients with SLE to take a maintenance dose of steroid during the perioperative period.

Furthermore, our study showed that SLE patients had a longer hospital stay. On the one hand, since SLE patients have progressively lower hemoglobin preoperatively and postoperatively, it may take them more time to improve their anemia and to mobilize their hematopoietic stem cells, partly explaining the extended length of hospital stay of the SLE patients. On the other hand, SLE patients underwent more aggressive treatments in the hospital, which costs more time. Similar findings have been published. For example, Kang et al. utilized a more aggressive prophylactic antibiotic regimen in total hip arthroplasty in patients with SLE [26]. In addition, Chen also reported that doctors and nurses spend more time and effort managing SLE patients, leading to no significant rise in the risk of perioperative adverse events in SLE patients [7].

A similar explanation may have to do with the consequences of perioperative adverse wound events, as some former studies have revealed that patients with SLE have a higher incisional infection rate [24, 27]. Nevertheless, in our research, SLE patients did not have a higher infection rate than controls. Interestingly, all of the adverse wound events in the control group were generated by swelling. However, SLE patients’ wounds are less likely to be swollen. Instead, other events, such as lipid liquefaction, happen, which may be associated with lower albumin levels or the use of steroids in SLE patients [28, 29]. Although the rate of postoperative albumin transfusions is also higher in patients with SLE, this outcome suggests that a more active preoperative albumin level increase may be of greater benefit to the postoperative incisional recovery of SLE patients.

This study suffers from the inherent weaknesses of retrospective investigations with small single-center samples, such as limited generalizability of the conclusions and the possible challenge in identifying variability among rare comorbidities. Another deficiency is the shortage of data from much earlier periods. Hence, we could not compare our outcomes with local center data from before the large-scale application of several accelerated rehabilitation models, such as TXA. However, this study’s data are based on detailed inpatient case histories, and more detail is available than in some recent large database studies. Our diagnostic and treatment processes were more precise, and there was less chance of data errors from repeated entry.

In summary, our data do not show that SLE patients have a significantly greater risk from THA than other patients, as long as they receive optimized surgical methods and perioperative management. SLE patients and others can achieve the same long-term functional recovery.

## Data Availability

Data used and analyzed in this study are available from the corresponding author on reasonable request.

## Abbreviations

SLE: Systemic lupus erythematosus
THA: Total hip arthroplasty;
Hb: Hemoglobin
Hct: Hematocrit
ESR: Erythrocyte sedimentation rate
CRP: C-reactive protein
Plt: Platelets
LOS: Length of stay
HHS: Harris Hip Score
SF-12: 12-item Short-Form Health Survey
TBL: Total blood loss
DVT: Deep vein thrombophlebitis
PE: Pulmonary embolism
